# Severity of Scorpion Stings in the Western Brazilian Amazon: A Case-Control Study

**DOI:** 10.1371/journal.pone.0128819

**Published:** 2015-06-10

**Authors:** Amanda M. Queiroz, Vanderson S. Sampaio, Iran Mendonça, Nelson F. Fé, Jacqueline Sachett, Luiz Carlos L. Ferreira, Esaú Feitosa, Fan Hui Wen, Marcus Lacerda, Wuelton Monteiro

**Affiliations:** 1 Departamento de Ensino e Pesquisa, Fundação de Medicina Tropical Doutor Heitor Vieira Dourado, Manaus, Brazil; 2 Escola Superior de Ciências da Saúde, Universidade do Estado do Amazonas, Manaus, Brazil; 3 Instituto Butantan, São Paulo, Brazil; 4 Instituto de Pesquisas Leônidas & Maria Deane (FIOCRUZ), Manaus, Brazil; Universidad de Costa Rica, COSTA RICA

## Abstract

**Background:**

Scorpion stings are a major public health problem in Brazil, with an increasing number of registered cases every year. Affecting mostly vulnerable populations, the phenomenon is not well described and is considered a neglected disease. In Brazil, the use of anti-venom formulations is provided free of charge. The associate scorpion sting case is subject to compulsory reporting. This paper describes the epidemiology and identifies factors associated with severity of scorpions stings in the state of Amazonas, in the Western Brazilian Amazon.

**Methodology/Principal Findings:**

This study included all cases of scorpion stings in the state of Amazonas reported to the Brazilian Diseases Surveillance System from January 1, 2007 to December 31, 2014. A case-control study was conducted to identify factors associated with scorpions sting severity. A total of 2,120 cases were reported during this period. The mean incidence rate in the Amazonas was 7.6 per 100,000 inhabitants/year. Scorpion stings showed a large spatial distribution in the state and represent a potential occupational health problem for rural populations. There was a positive correlation between the absolute number of cases and the altimetric river levels in the Central (p<0.001; R_s_ = 0.479 linear) and Southwest (p = 0.032; linear R_s_ = 0.261) regions of the state. Cases were mostly classified as mild (68.6%), followed by moderate (26.8%), and severe (4.6%). The overall lethality rate was 0.3%. Lethality rate among children ≤10 years was 1.3%. Age <10 years [OR = 2.58 (95%CI = 1.47–4.55; p = 0.001)], stings occurring in the rural area [OR = 1.97 (95%CI = 1.18–3.29; p = 0.033) and in the South region of the state [OR = 1.85 (95%CI = 1.17–2.93; p = 0.008)] were independently associated with the risk of developing severity.

**Conclusions/Significance:**

Scorpion stings show an extensive distribution in the Western Brazilian Amazon threatening especially rural populations, children ≤10 in particular. Thus, the mapping of scorpions fauna in different Amazon localities is essential and must be accompanied by the characterization of the main biological activities of the venoms. Urban and farming planning, in parallel with awareness of workers at risk for scorpion stings on the need for personal protective equipment use should be considered as public policies for preventing scorpionism.

## Introduction

Scorpion stings are a major health problem, notably in developing countries. The African continent, the Middle East, Southern India and Latin America, especially Mexico, Brazil and other countries of the Amazon region (Guianas and Venezuela) are the locations where they show the highest incidence and/or severe cases. Approximately two billion people live in at risk areas for scorpion envenomation incidents; these accidents occur at a frequency of over a million annually worldwide [1]. Scorpion venoms cause the release of autonomic nervous system mediators causing myocardial damage, cardiac arrhythmias, pulmonary edema and shock. Stimulation of the peripheral nervous system results in uncoordinated neuromuscular activity, manifested as muscle spasms, dysmetria and dysarthria [2,3]. Early administration of antivenom is highly effective, together with intensive care support in severe cases. However, the rapid tissue distribution of scorpion venom toxins and their ability to cause early death demand early antivenom treatment and full cardio-respiratory support [4].

Countries with tropical and subtropical climates are places where scorpion stings most commonly occur [5], especially in the warmer months [6,7]. High temperatures make scorpions more active and therefore increase their proximity to humans [8]**.** The incidence of scorpion stings is directly affected by the rain, especially heavy rain, they show a higher occurrence in rainy months [9]**,** probably due to rainfall flooding the natural habitats of scorpions and forcing them to seek refuge [10].

Scorpion stings are an emerging and neglected public health problem in Brazil, with an increasing number of registrations every year in the country and 78,091 cases reported in 2013 [11]. In Brazil, the main species of medical interest belongs to the genus *Tityus*, which has a high adaptive capacity in anthropic environments [12]. Outside the Amazon, *T*. *serrulatus* is the major causative agent of scorpionism, resulting in high severity and lethality rates [11,12]. The major species of medical interest in the Brazilian Amazon are *T*. *obscurus*, *T*. *metuendus* and *T*. *silvestris*. Scorpion stings in this region are mostly caused by *T*. *obscurus*, especially in the state of Pará, which is the main cause of accidents, having the potential to cause serious and fatal accidents [3,13]. In the state of Amazonas, Western Brazilian Amazon, a clinical-epidemiological study found that the majority of reported accidents were caused by *T*. *metuendus*, including fatalities [14]. A study carried out in the state of Acre found that 10% of tappers and 14% of Amerindians had been stung by scorpions at least once in their lifetime [15].

This study was aimed at describing the epidemiology and identifying risk factors for severity of scorpions stings in the Brazilian state of Amazonas (western area), still with very low deforestation, based on reliable surveillance records.

## Methods

### Ethical clearance

This study was approved by the Ethics Review Board (ERB) of the *Fundação de Medicina Tropical Dr*. *Heitor Vieira Dourado* (approval number 872.520/2014). All data analyzed were anonymized. Since data were obtained exclusively from surveillance databases, the ERB gave a waiver of informed consent.

### Study area

The State of Amazonas is located in the western Brazilian Amazon (latitude 2°01′ N, longitude 73°48′ W), comprising an area of 1,570,946.8 km^2^, with four major regions (North, South, Central and Southwestern) and 62 municipalities. The estimated population of the state was 3,807,921 inhabitants in 2010, with 74.2% living in the urban zones and 25.8% in rural areas. Vegetation is mainly a dense evergreen rain forest. Climate is classified as the equatorial super-humid type, with rainfalls over 2,000 mm *per annum* and average annual temperatures between 26°C and 28°C. There is no clear distinction between dry and rainy seasons and the temperatures present a little variation throughout the state area [16]. Most of the Gross Domestic Product in the state is based on the industrial sector in the capital Manaus, which hosts a large number of national and multinational companies. Within the state are increasing investments in areas such as fish farming, agribusiness and rural production. In the South of the state, the huge increase in cattle, logging and subsistence farming (most recently mechanized cultivation of soybeans and cotton), has led to deforestation of large areas.

### Study subjects

Scorpion stings are compulsorily recorded by the Brazilian Notifiable Diseases Information System [*Sistema de Informação de Agravos de Notificação* (SINAN)] based on data from forms used in the investigation and follow-up of cases of animal envenomings. SINAN is the main survey system for collection and analysis of national data on scorpion stings in Brazil [17]. This study was performed including all cases of scorpion stings in the state of Amazonas reported to SINAN from January 1, 2007 to December 31, 2014.

### Study design and definitions

We performed a descriptive analysis of the absolute and relative frequencies of the characteristics of the patients presenting with scorpion stings to ensure that the distributional assumptions for statistical tests were met. The variables analyzed were sex, age (in years), anatomical region of the bite, area of occurrence (rural or urban), work-related injury (yes or no), time elapsed between the bite and medical assistance (in hours), severity grading (mild, moderate or severe) and outcome (discharge or death). All variables were checked for duplicity and completeness by two independent researchers before analysis and further investigated for a possible association with severity and as dependent variables.

A map was created with the software ArcMap 10.1 in ArcGIS 10.1 (ESRI, USA) using estimates of the mean incidence by municipality. The incidence rate was calculated by dividing cases by the population of each municipality in the middle of the period [18] by 100 thousand inhabitants. Spatial interpolation of scorpion stings incidence was mapped using data from 62 municipalities by the Inverse Distance Weighting (IDW) method. For this analysis, the output cell size was set to 0.33, a power value of 2 was used, the search neighbourhood was set to smooth circular with a 30.15 radius and a smoothing factor of 0.2 was used. No features were used as barriers.

Information about scorpion stings severity is a required field on the SINAN survey system. This information is collected from medical records by local surveillance professionals, after the diagnosis has been confirmed. Clinical severity of scorpion stings was classified according to the Brazilian Ministry of Health guidelines. Although this classification has not undergone validation studies it is based on the consensus of national specialists [19] and in agreement with international experts [20]: i) A mild case was defined as a scorpion sting with local pain and paresthesia; ii) A moderate case was defined as a scorpion sting with intense local pain associated with one or more systemic manifestations, including nausea, vomiting, sweating, mild drooling, restlessness, tachypnea and tachycardia; iii) A severe case was defined as a scorpion sting presenting the same symptoms as moderate cases, but evolving to profuse and uncontrollable vomiting, profuse sweating, severe drooling, prostration, convulsions, coma, bradycardia, heart failure, acute pulmonary edema and shock.

A case-control study was performed to identify factors associated with scorpion sting severity. Cases and controls groups were selected considering their classification as outlined above. Severe stings were included in the group of cases and mild and moderate stings were included in the control group.

### Statistical analysis

Only variables presenting completeness higher than 80% were analyzed as factors related to severity. Data were analyzed using SPSS version 21.0 for Windows (SPSS Inc. Chicago, IL, USA). The annual incidence was calculated using the average of cases in six years and the population at risk in the middle of the period for the state of Amazonas and in each municipality, for 100,000 inhabitants. A Chi-square test for trend was performed to test the increase of the incidence rate during the study period. Proportions of severe cases were compared by Chi-square test (corrected by Fisher's test if necessary). Normal distribution of data was evaluated with the Kolmogorov-Smirnov test. Non-parametric Spearman's test was used to assess the correlation between the absolute number of cases and the altimetric river levels. The crude *Odds Ratio* (OR) with its respective 95% Confidence Interval (95% CI) was determined considering the severity as the dependent variable. Logistic regression was used for the multivariate analyses and the adjusted ORs with 95% CI were also calculated. A multivariate logistic regression was performed with scorpion stings severity as the outcome, using an automated backward and forward stepwise estimation. All variables that were associated with severity at a significance level of p<0.20 in the univariate analysis were included in the multivariate analysis. Model calibration was evaluated using the Hosmer-Lemeshow goodness-of-fit test. Statistical significance was considered if p<0.05.

## Results

### General characteristics

Table 1 shows the general characteristics of scorpion stings in the State of Amazonas, from 2007 to 2014. According to the official reporting systems, during the period there were 2,120 cases of scorpion stings in the State of Amazonas. Most of these occurred in males (63.9%). Regarding the area of occurrence, 56.6% were reported from rural areas. A proportion of 38.7% of the stings were classified as work related accidents. The most common occupation group of the victims was farmer/fisher (72.4%). The most affected age group was between 21 and 30 years old (19.7%). An admixed ethnical background was predominant (76.3%). Stings occurred mostly in the upper (47.9%) and lower limbs (46.5%) respectively. The majority of the cases (69.6%) received medical assistance within the first 3 hours after the sting.

**Table 1:** Characteristics of the 2,120 scorpion stings reported in the State of Amazonas, 2007 to 2014.

Characteristics (completeness)	Number	%
**Sex (n = 2,120; 100%)**		
Male	1,354	63.9
Female	766	36.1
**Area of occurrence (n = 2,076; 97.9%)**		
Rural	1,176	56.6
Urban	900	43.4
**Age group in years (n = 2,120; 100%)**		
0-10	313	14.8
11-20	395	18.6
21-30	418	19.7
31-40	385	18.1
41-50	313	14.8
51-60	174	8.2
≥61	122	5.7
**Ethnicity (n = 2,065; 97.4%)**		
Admixed	1,575	76.3
White	229	11.1
Indian	192	9.3
Black	58	2.8
Asian	11	0.5
**Work related accident (1,905; 89.9%)**		
Yes	737	38.7
No	1,168	61.3
**Occupation (n = 751; 35.4%)**		
Farmer/Fisher	544	72.4
Trade and services employee	134	17.8
Industry employee	44	5.9
Other	29	3.9
**Anatomical region of the sting (n = 2,042; 96.3%)**		
Head	47	2.3
Upper limbs	979	47.9
Body	67	3.3
Lower limbs	949	46.5
**Time elapsed from sting to medical assistance (hrs) (n = 1,997; 94.2%)**		
0-3	1,391	69.6
4-6	341	17.1
7-12	139	6.9
13-24	69	3.4
>24	57	2.8
**Case severity (n = 2,047; 96.5%)**		
Mild	1,404	68.6
Moderate	549	26.8
Severe	94	4.6
**Outcome (n = 2,120; 100%)**		
Discharged	2,114	99.7
Death	6	0.3

Cases were mostly classified as mild (68.6%), followed by moderate (26.8%) and severe (4.6%) cases. There were 6 deaths due to scorpion stings in the study period, resulting in a 0.3% lethality rate. Four victims were children ≤10 years of age, with a lethality rate of 1.3% in this age group.

**Fig 1:**
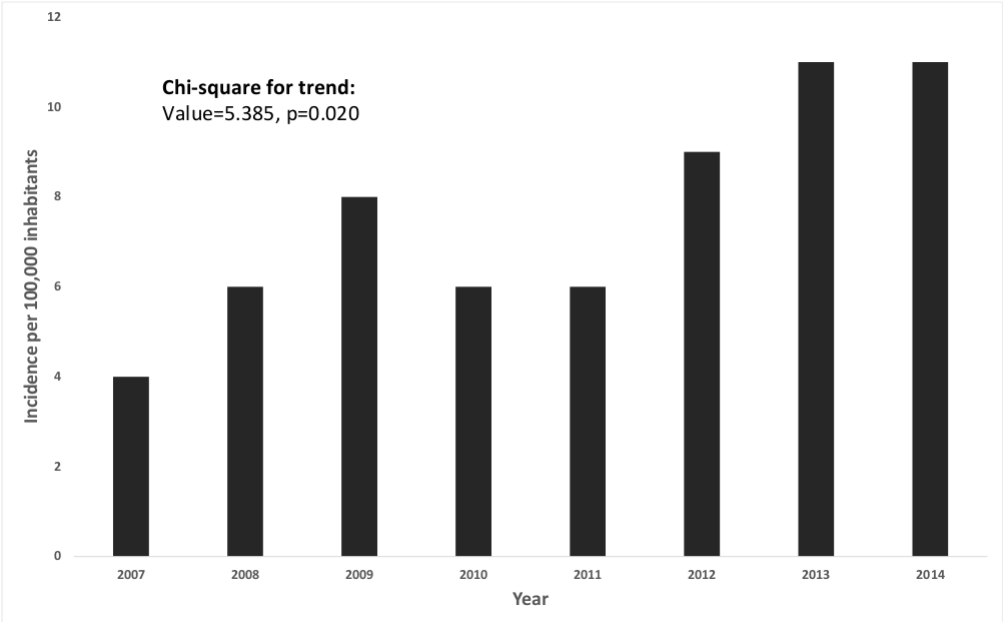
Annual incidence of scorpionism on Amazonas, from 2007 to 2014. Incidence was expressed as the number of cases per 100,000 inhabitants. In 2007 the incidence was 4/100,000, increasing to 11/100,000 inhabitants in 2014.

There was an increase in incidence rates during the study period (Chi-square test for trend; p = 0.020). In 2007 the incidence was 4 per 100,000, increasing to 11 cases per 100,000 inhabitants in 2014 (Fig 1).

### Spatial distribution

**Fig 2:**
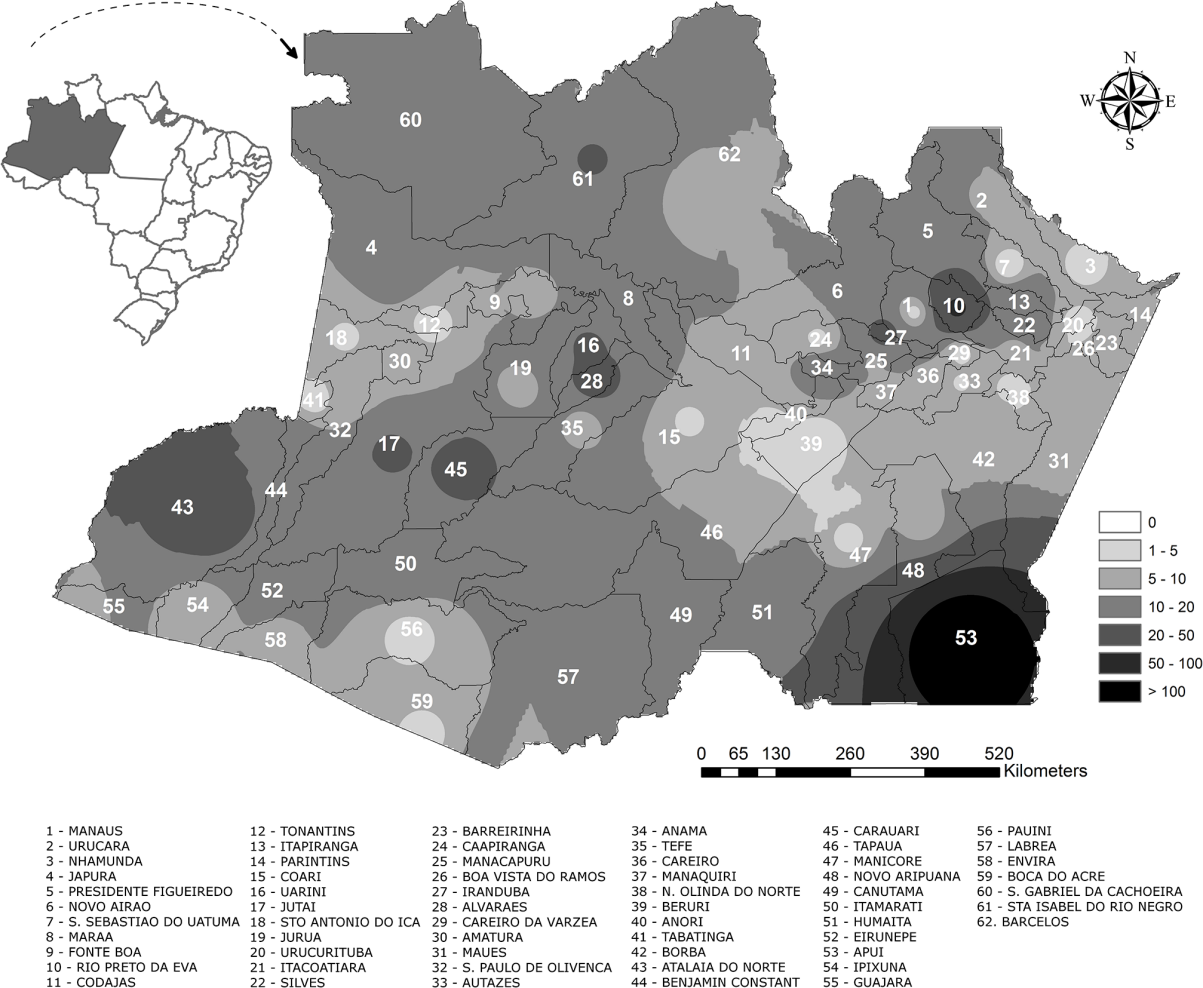
Spatial distribution of scorpion stings in the State of Amazonas, from 2007 to 2014. Incidence rates were unevenly distributed across the Amazonas State. Extensive hot spots are shown in the South Region, as well as in Manaus surroundings, (municipality of Rio Preto da Eva) with incidence rates >50/100,000/year.

The mean incidence rate in the Amazonas State was 7.6 per 100,000 inhabitants/year. Mapping showed a large swath in the South Region where scorpionism was predicted to have incidence rates >50 cases/100,000 inhabitants/year. Incidence rates were also higher >50 cases/100,000 inhabitants/year in Manaus surroundings, namely in the municipality of Rio Preto da Eva (Fig 2). Incidence rates were unevenly distributed across the Amazonas State, although there were scorpion stings cases reported from 61 (98.4%) out 62 municipalities. The municipality with the highest mean incidence was Apuí with 183.8 cases per 100,000 inhabitants/year. Rio Preto da Eva was the second municipality in number of cases, with an mean incidence of 58.9 per 100,000 inhabitants/year (S1 Table).

### Seasonality

**Fig 3:**
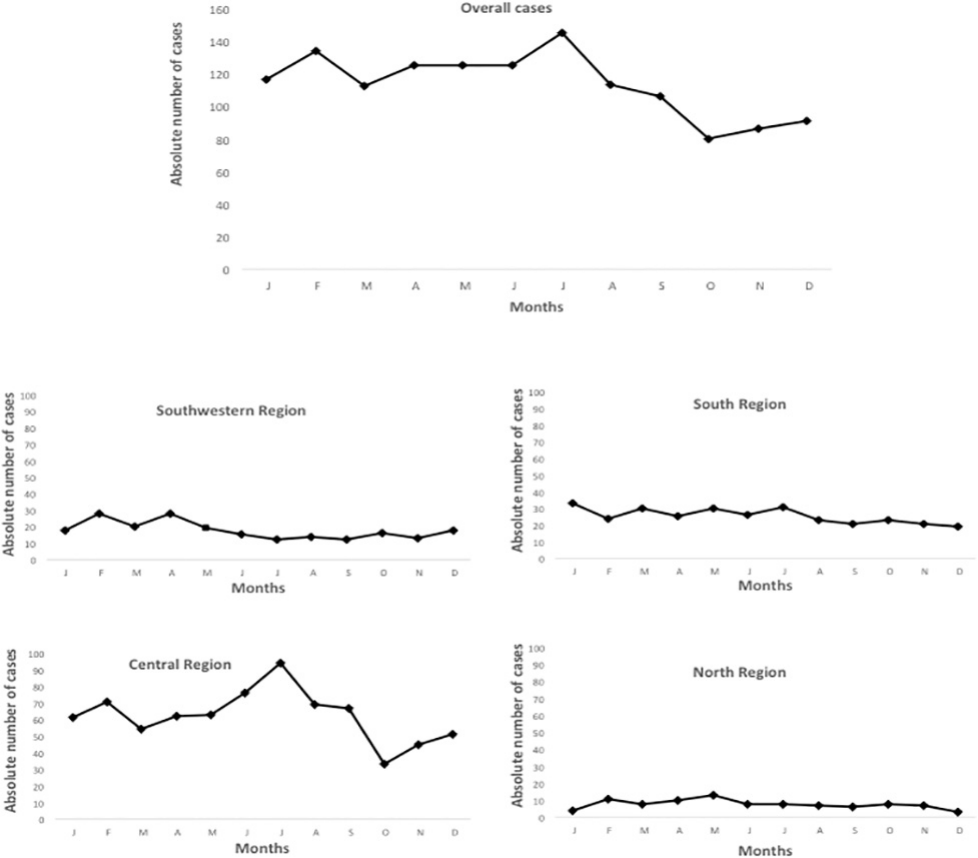
Seasonality of the scorpion stings reported in the State of Amazonas, by region, from 2007 to 2014. An increase in the number of scorpion stings in the Amazonas between June and July is seen, as well as in the Central Region of the state. In the Southwest Region, the number of cases is higher between February and May. In the South and North regions seasonality was no pronounced.

There was an increase in the number of scorpion stings in the Amazonas between June and July. The same was observed in the Central region of the state. In the Southwest region, the number of cases is higher between February and May, correlating with the rainy season. In South and North regions seasonality was no pronounced (Fig 3).

**Fig 4:**
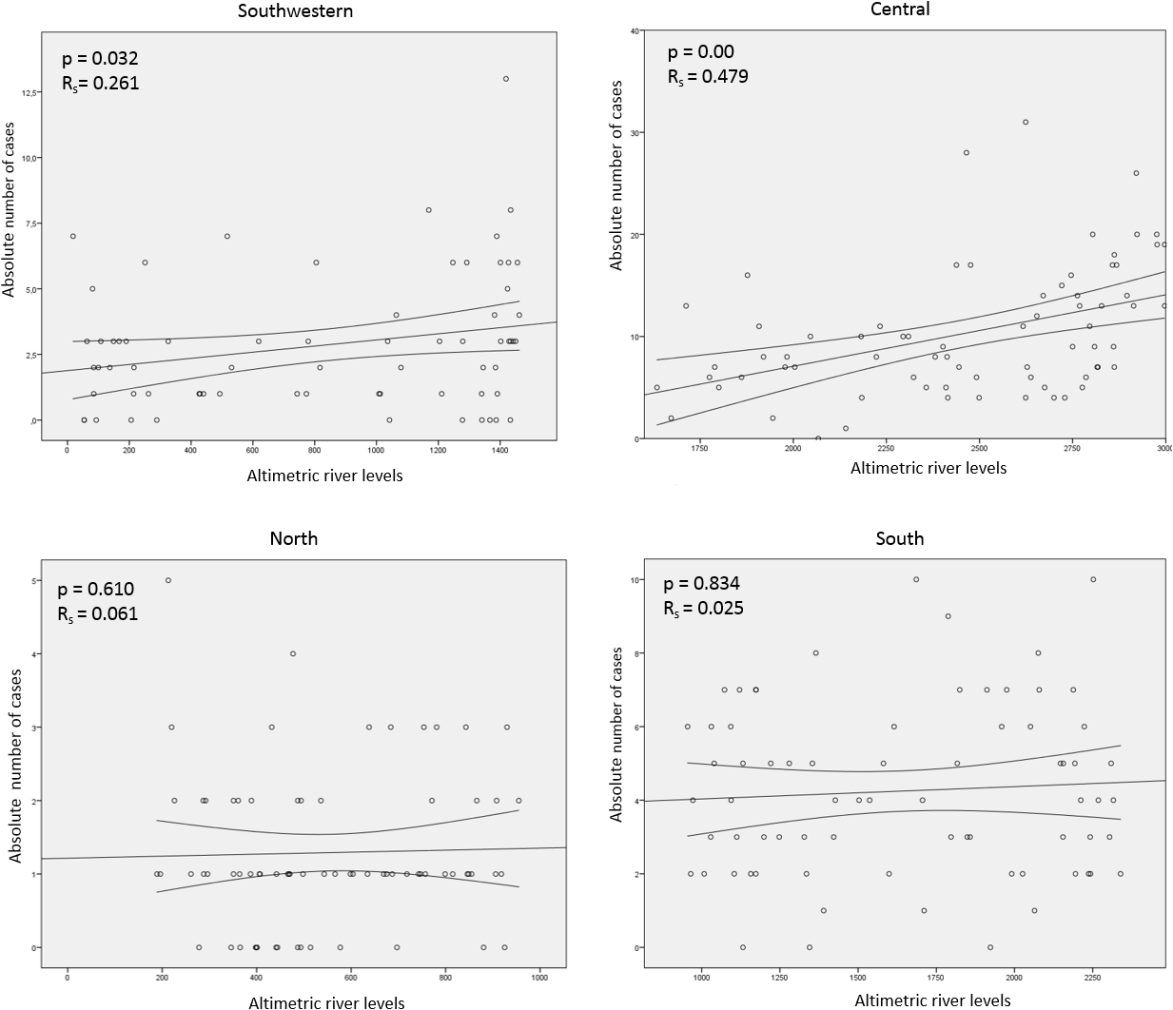
Scorpion stings and altimetric river levels in the State of Amazonas, 2007 to 2014. A correlation between the absolute number of cases and the altimetric river levels was notable in the Central and Southwest regions, in contrast to the South and North regions where this correlation was not observed.

There was a correlation between the absolute number of cases and the altimetric river levels in the Central (p<0.001; R_s_ = 0.479 linear) and Southwest (p = 0.032; linear R_s_ = 0.261) regions. In the South (p = 0.834; linear R_s_ = 0.025) and North (p = 0.610; linear R_s_ = 0.061) regions this correlation was not observed (Fig 4).

### Factors associated to severity

**Fig 5:**
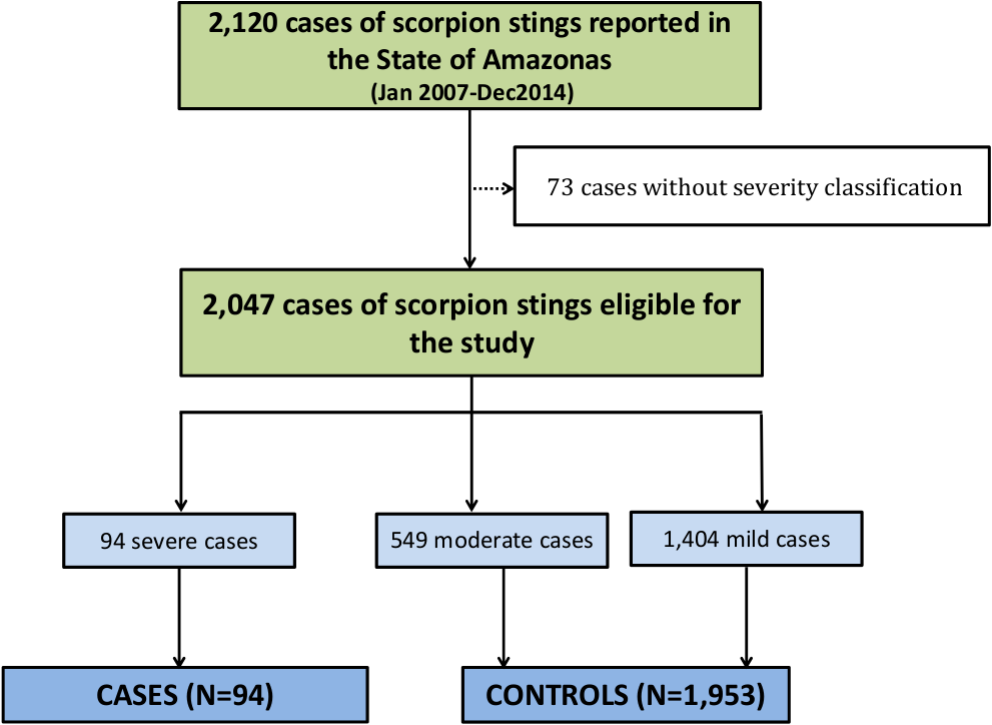
Flow chart of cases and control selection. The selection of cases and controls groups was based on the Brazilian Ministry of Health classification. All severe stings were included as cases, and three mild and moderate stings were in the control group for each case.

For 94 cases of severe scorpion stings during the study period, 1,953 controls were selected among mild and moderate cases (Fig 5). Table 2 summarizes the results of the univariate and multivariate logistic regression evaluating factors associated with scorpion stings severity. Age <10 years [OR = 2.58 (95%CI = 1.47–4.55; p = 0.001)], stings occurring in the rural area [OR = 1.97 (95%CI = 1.18–3.29; p = 0.033) and in the South region of the state [OR = 1.85 (95%CI = 1.17–2.93; p = 0.008)] were independently associated with the risk of developing severity.

**Table 2:** Factors associated with scorpion stings severity in the State of Amazonas, 2007 to 2014.

Variables	Cases (n)	%	Controls (n)	%	Crude odds ratio (IC 95%)	p	Adjusted odds ratio (IC 95%)	p
**Sex**								
Male	66	70.2	1,239	74.8	**1.36 (0.86-2.13)**	**0.182**	1.08 (0.67-1.75)	0.754
Female	28	29.8	714	25.2				
**Age (years)**								
0-10	72	76.6	1,681	86.1	**1.88 (1.15-3.09)**	**0.003**	**2.58 (1.47-4.55)**	**0.001**
>10	22	23.4	272	13.9				
**Area of occurrence**								
Rural	71	75.5	1,064	55.5	**2.47 (1.54-4.00)**	**<0.001**	**1.97 (1.18-3.29)**	**0.033**
Urban	23	24.5	854	44.5				
**Work related accident**								
Yes	47	54.0	673	38.0	**1.92 (1.24-2.96)**	**0.009**	0.71 (0.29-1,73)	0.110
No	40	46.0	1,099	62.0				
**Ethnicity**								
Admixed	70	76.1	1,460	76.5		0.468	…	…
White	12	13.1	212	11.1				
Indian	5	5.4	175	9.2				
Black	5	5.4	51	2.7				
Asian	0	-	11	0.6				
**Anatomical site**								
Head	5	5.3	39	2.1		**0.123**	…	…
Upper limbs	48	51.1	899	47.8				
Body	4	4.2	62	3.3				
Lower limbs	37	39.4	882	46.8				
Head	5	5.3	39	2.1	**2.65 (1.02-6.89)**	**0.037**	0.66 (0.38-1.13)	0.126
Other sites	89	94.7	1,843	97.9				
**Time until medical assistance (hrs)**								
0-3	60	65.9	1,294	69.8	0.84 (0.54-1.31)	0.439	…	…
>3	31	34.1	561	30.2				
**Region of the state**								
South	35	37.2	404	20.7		**<0.001**	…	…
Central	36	38.3	1,156	59.2				
Southeasthern	22	23.4	278	14.2				
North	1	1.1	115	5.9				
South	35	37.2	404	20.7	**2.27 (1.48-3.50)**	**<0.001**	**1.85 (1.17-2.93)**	**0.008**
Other regions	59	62.8	1,549	79.3				

## Discussion

In this study, an extensive distribution of scorpion stings in the state of Amazonas was revealed, where a trend of increasing incidence over the study period was notable. Case distribution was heterogeneous, with a hot spot observed in the southern state, especially in Apuí, where the average annual incidence reached more than 180 cases per 100,000 inhabitants/year, coinciding with the areas of farming expansion from the southern country. In this region, there is a huge recent increase of cattle, logging and farming activities, including most recently mechanized cultivation of soybeans and cotton. In this context, the destruction of the natural habitat of scorpions could lead to increased contact between these animals and humans during work activities. This is of concern for the sustainable development of the state, especially regarding forest preservation parallel with reasonable quality of life for the population. The incidence in this municipality is comparable to the effects found in regions of Iran [7], Colombia [21], Turkey [22] and other regions of Brazil [23,24], where scorpion stings are also a major public health problem.

We emphasized that scorpion stings are an important occupational health hazard in rural areas of the Amazon, affecting mostly males of working age. In some large natural regions such as the Amazon, human populations have remained in low numbers for centuries, and only in recent years a rapid expansion has occurred. Isolated fatal cases have been observed after *Tityus metuendus* e *Tityus obscurus* stings. These are probably equilibrium species and will be partially or totally selected against by the destruction of their environment before human populations become large and vulnerable to them [24]. The ends of the upper and lower limbs were the most affected anatomical regions, totaling 94.4% of cases, in agreement with previous results from the literature [25–27]. As a preventive measure the protection of these body with the use of gloves and boots for populations at high risk during work is suggested. Workers’ health in this region needs more attention from policy-makers. A substantial number of patients with mild bites in remote rural areas may not report to hospitals, explaining the higher severity for accidents recorded from rural locations.

The time between the accident and medical assistance in the state, as reported also in other regions of Brazil [28], was less than 3 hours in 69.6% of cases. Most of the severe cases (79.4%) also received treatment in less than 6 hours after the sting, probably contributing to preventing higher lethality in the Amazon. This may be related to the location of most cases in urban areas or rural areas in the southern region of the state with roads and less isolated human settings which would facilitate access to specialized medical treatment.

There was a positive correlation between the absolute number of cases and the altimetric river level in the central and Southwest regions. This correlation is probably due to the fact that most rainfall floods the natural habitat of scorpions, forcing them to seek new shelters [9,10]. In the Southeast and Northeast of Brazil, most accidents occur during the warm and rainy months [8,23,25,29]. This association was also suggested in French Guiana [30], Mexico [10], Venezuela [31] and West Africa [9]. In the South and North regions of the State of Amazonas this correlation was not observed. In the North region, cases were scarce and sparsely distributed, specially among Amerindian villages. In the South region seasonality was not observed, which can be explained by the location of the municipality of Apuí (where the majority of cases was recorded), far from the river banks, thus not subjected to floods. In this region, non-seasonal agricultural activities may be related to scorpion stings incidence. The influence of temperature in the scorpion activity and scorpion sting incidence has been documented [32,33], but socioeconomic factors should be considered, as observed in this study.

Age ≤10 years was independently associated with the risk of developing life-threatening complications. Scorpion stings show greater severity in childhood, suggesting that there is a direct relationship between venom levels in the plasma and the poisoning severity [34] by which venom would reach higher plasma concentrations in children due to their lower body mass [9]. Similar results were found in other parts of Brazil [35,36], Mexico [10], Colombia [37] and Argentina [38]. In this study, lethality rate among children ≤10 years was 1.3%, four times higher than in the general population, showing scorpion stings as a cause of death among children in the Brazilian Amazon. A high lethality rate due to scorpion stings may result from challenges found in small towns in the Amazon related to: experience of health personnel, supply of appropriate antivenom therapy and quality of care, with the latter being dependent on equipment from health facilities (resuscitation equipment particularly) [4]. Therefore, in these small Amazon towns located far from reference centers, investment in training health professionals in the initial management of the patient and follow up of possible complications of scorpion stings is essential.

There are two types of antivenoms available in Brazil for scorpion stings: *Tityus* scorpion antivenom and a polyvalent antivenom against spiders (*Loxosceles* and *Phoneutria*) and *Tityus* scorpions. Scorpion antivenom is produced by immunizing horses with *Tityus serrulatus* antigen, whereas other *Tityus* species are the prevalent scorpions in the Amazon region. However, some evidence suggests toxicity variation resulting from the diversity of *T*. *obscurus* venom in different Amazon areas [39]. Thus, new studies and investment in technological development are needed to assess different antivenom candidate formulations in the Amazon [40].

Interestingly, scorpion stings occuring in the South region of the state were also independently associated with severity. Unfortunately, there is scarce information on scorpion fauna in this region, but a plausible explanation may be due to the toxicity of the venom of species of *Tityus* spp. present in the area. Sufficient taxonomical and distribution information is available for thirteen *Tityus* spp. reported for the state of Amazonas [41,42]. *Tityus silvestris* has an extensive geographic distribution, including a large swath in the Southern Amazonas, and it was implicated as a scorpionism agent in the state of Pará [43]. In the Manaus region, accidents were mostly caused by *T*. *metuendus*, including fatalities [14]. *Tityus obscurus* has been shown in the State of Pará causing severity in stings occurring in the western state [3,13], a region that borders the South of the Amazonas. It is noteworthy that in SINAN reporting forms there is no mention of scorpions species involved in the accidents. Thus, the identification of the scorpion species is essential to guide future clinical studies and antivenom testing in the area.

Record keeping may have been influenced by the nature of the surveillance system. Some patients with mild bites in inaccessible areas may not report to hospitals and those evolving to severity may die on the way before reaching medical attention. Another limitation of this study was related to the lack of standardization in data collection in different regions of the state and the lack of information such as the scorpion species causing the stings. However, the broad population coverage and the low cost for data collection allowed obtaining valuable information. Because scorpion stings are of compulsory report, independently of specific antivenom therapy, statistics of scorpionism in SINAN is believed to be reliable and reasonably complete in Brazil. We highlight that estimates of independent risk factors for complications should be confirmed by means of multicenter prospective studies.

In conclusion, scorpion stings showed a extensive distribution in the Western Brazilian Amazon and represent a potential occupational health problem for rural populations in this region. Correlation between the absolute number of cases and the altimetric river levels in some regions of the state highlight vulnerability of riverine Amazonian communities living in river bank areas. Age ≤10 years and stings occuring in the South region of the state were independently associated with the risk of developing severity, demanding a response from surveillance system and policy makers for these populations. Thus, the mapping of scorpions fauna in different Amazon localities is essential and must be accompanied by the characterization of the main biological activities of the venoms. The planning of urbanization and farming activities, in parallel with the awareness of workers at risk for scorpion stings on the need for personal protective equipment use should be considered as public policies for preventing scorpionism.

## Supporting Information

S1 File STROBE Statement: Checklist of items that should be included in reports of case-control studies. (DOC)

S1 Table Annual mean incidence by municipality in the State of Amazonas, 2007–2014. (DOC)
