# Supplementary material for: Severity of Scorpion Stings in the Western Brazilian Amazon: A Case-Control Study
Source: PLoS One. 2015 Jun 10;10(6):e0128819. doi: 10.1371/journal.pone.0128819 (PMC4465172; doi:10.1371/journal.pone.0128819)
Supplement: S1 Table — (DOC) [file pone.0128819.s002.doc]

**Table S1.** Annual mean incidence by municipality in the State of Amazonas, 2007-2014.

| **Municipality** | **Population** | **Number of cases** | **Mean incidence** | **Municipality** | **Population** | **Number of cases** | **Mean incidence** |
| --- | --- | --- | --- | --- | --- | --- | --- |
| **Alvarães** | 14,381 | 31 | 26.9 | **Japurá** | 7,448 | 10 | 16.8 |
| **Amaturá** | 9,794 | 6 | 7.7 | **Juruá** | 11,439 | 7 | 7.6 |
| **Anamã** | 10,766 | 16 | 18.6 | **Jutaí** | 18,293 | 34 | 23.2 |
| **Anori** | 17,072 | 4 | 2.9 | **Lábrea** | 39,022 | 48 | 15.4 |
| **Apuí** | 18,633 | 274 | 183.8 | **Manacapuru** | 86,985 | 62 | 8.9 |
| **Atalaia do Norte** | 15,924 | 46 | 36.1 | **Manaquiri** | 24,325 | 15 | 7.7 |
| **Autazes** | 33,312 | 11 | 4.1 | **Manaus** | 1,861,838 | 450 | 3.0 |
| **Barcelos** | 25,948 | 12 | 5.8 | **Manicoré** | 48,373 | 12 | 3.1 |
| **Barreirinha** | 28,077 | 13 | 5.8 | **Maraã** | 17,596 | 18 | 12.8 |
| **Benjamin Constant** | 34,950 | 49 | 17.5 | **Maués** | 54,079 | 34 | 7.9 |
| **Beruri** | 16,158 | 2 | 1.5 | **Nhamundá** | 18,720 | 3 | 2.0 |
| **Boa Vista do Ramos** | 15,659 | 6 | 4.8 | **Nova Olinda do Norte** | 31,749 | 6 | 2.4 |
| **Boca do Acre** | 31,171 | 8 | 3.2 | **Novo Airão** | 15,489 | 14 | 11.3 |
| **Borba** | 35,919 | 20 | 7.0 | **Novo Aripuanã** | 22,106 | 10 | 5.6 |
| **Caapiranga** | 11,303 | 3 | 3.3 | **Parintins** | 103,828 | 124 | 14.9 |
| **Canutama** | 13,986 | 22 | 19.7 | **Pauini** | 18,329 | 3 | 2.1 |
| **Carauari** | 26,130 | 54 | 25.8 | **Presidente Figueiredo** | 28,652 | 29 | 12.6 |
| **Careiro** | 33,517 | 19 | 7.1 | **Rio Preto da Eva** | 26,948 | 127 | 58.9 |
| **Careiro da Várzea** | 24,937 | 5 | 2.5 | **Santa Isabel do Rio Negro** | 19,292 | 32 | 20.7 |
| **Coari** | 77,305 | 25 | 4.0 | **Santo Antônio do Içá** | 24,890 | 8 | 4.0 |
| **Codajás** | 24,067 | 11 | 5.7 | **São Gabriel da Cachoeira** | 39,097 | 33 | 10.5 |
| **Eirunepé** | 31,364 | 30 | 12.0 | **São Paulo de Olivença** | 32,677 | 15 | 5.7 |
| **Envira** | 16,923 | 12 | 8.9 | **São Sebastião do Uatumã** | 11,241 | 0 | 0 |
| **Fonte Boa** | 23,198 | 14 | 7.5 | **Silves** | 8,544 | 14 | 20.5 |
| **Guajará** | 14,396 | 7 | 6.1 | **Tabatinga** | 54,440 | 10 | 2.3 |
| **Humaitá** | 45,954 | 39 | 10.6 | **Tapauá** | 17,903 | 19 | 13.3 |
| **Ipixuna** | 23,460 | 11 | 5.9 | **Tefé** | 61,000 | 34 | 7.0 |
| **Iranduba** | 41,947 | 90 | 26.8 | **Tonantins** | 17,316 | 3 | 2.2 |
| **Itacoatiara** | 89,064 | 48 | 6.7 | **Uarini** | 12,139 | 21 | 21.6 |
| **Itamarati** | 7,983 | 9 | 14.1 | **Urucará** | 16,902 | 12 | 8.9 |
| **Itapiranga** | 8,348 | 13 | 19.5 | **Urucurituba** | 18,679 | 3 | 2.0 |
